# Variations in Subscapularis Muscle Innervation—A Report on Case Series

**DOI:** 10.3390/medicina56100532

**Published:** 2020-10-12

**Authors:** Martin Siwetz, Niels Hammer, Benjamin Ondruschka, David C. Kieser

**Affiliations:** 1 Department of Macroscopic and Clinical Anatomy, Medical University of Graz, 8010 Graz, Austria; martin.siwetz@medunigraz.at; 2 Fraunhofer IWU, Medical Branch, 01187 Dresden, Germany; 3 Department of Orthopedic and Trauma Surgery, University of Leipzig, 04109 Leipzig, Germany; 4 Institute of Legal Medicine, University Medical Center Hamburg-Eppendorf, 22529 Hamburg, Germany; b.ondruschka@uke.de; 5 Department of Orthopaedic Surgery and Musculoskeletal Medicine, University of Otago, 8140 Christchurch, New Zealand; kieserdavid@gmail.com

**Keywords:** anatomical variation, lower subscapular nerve, subscapularis, muscle innervation, upper subscapular nerve

## Abstract

*Background and objectives:* The subscapularis muscle is typically innervated by two distinct nerve branches, namely the upper and lower subscapular nerve. These usually originate from the posterior cord of the brachial plexus. A large number of variations have been described in previous literature. *Materials and Methods:* Dissection was carried out in 31 cadaveric specimens. The frequency of accessory subscapular nerves was assessed and the distance from the insertion points of these nerves to the myotendinous junction was measured. *Results:* Accessory subscapular nerves were found in three cases (9.7%). According to their origin from the posterior cord of the brachial plexus proximal to the thoracodorsal nerve all three nerves were identified as accessory upper subscapular nerves. No accessory lower subscapular nerves were found. *Conclusion:* Accessory nerves occur rather commonly and need to be considered during surgery, nerve blocks, and imaging procedures.

## 1. Introduction

The brachial plexus is typically formed by the ventral rami of the lower four cervical and the upper thoracic nerve roots. These ventral rami fuse to build an upper, middle, and lower trunk, which subsequently split into anterior and posterior divisions and then three cords, namely a medial, lateral, and posterior cord. Chaudhary and colleagues described that, in 95% of specimens, the posterior cord is formed by the union of the posterior divisions of the three trunks, while in the remaining 5%, another (fourth) trunk exists [1].

The subscapularis muscle originates from the subscapular fossa of the scapula and inserts as a proximal tendinous and a distal muscular part at the lesser tuberosity and the humeral neck distal to the lesser tuberosity [2]. In rare cases an accessory subscapularis muscle can be found [3]. The subscapularis muscle is typically innervated by two distinct nerves, namely an upper and a lower subscapular nerve, both originating from the brachial plexus [4]. These two nerve branches cross the axillary vein dorsally to reach the subscapularis muscle [5]. Bergmann and colleagues reported that either the C5 and C6 nerve roots, or a combination of both may form the upper subscapular nerve. Beyond this, C4 may also contribute to the upper subscapular nerve [6]. The typical pattern described in textbooks are two subscapular nerves, both originating from the posterior cord of the brachial plexus [7]. Nevertheless, the existing literature describes the subscapular nerves as being rather variable in their origin [1,4,8,9,10,11,12,13,14,15,16] and there are some reports about accessory subscapular nerves [8,10,11,12,14,15,16].

This given case series aimed to provide data on the anatomical variations, with focus on accessory nerves. These accessory nerves need to be considered especially during surgery in the shoulder and axilla area as well as nerve blocks and imaging procedures.

## 2. Materials and Methods

This given case series analyzed 31 human shoulders (15 right, 16 left, 10 females, 21 males, age range 55 to 95 years). Ethical approval was obtained from the ethical review committee of the Medical University of Graz (approval number 32-282 ex 19/20, date of issue 5 May 2020). Samples were included only if they had no signs of pathologic lesions, major traumatic injury, or post-operative sequalae in the neck and shoulder regions. All samples were embalmed using the Thiel embalm method [17,18]. Dissection involved approaching the plexus from above, via the deltopectoral groove and from below via the axillary fossa.

First an incision was made over the deltopectoral grove. Skin and subcutaneous tissue were removed medially and laterally. The pectoralis major muscle was exposed and detached from its insertion and its clavicular origin to improve visibility. The clavipectoral fascia and fatty tissue was removed to gain view onto the brachial plexus. Further dissection was carried out from below through the axilla where skin, fatty tissue, and axillary fascia were removed. The brachial plexus was dissected free from two directions and the path and distribution of nerves innervating the subscapularis were investigated from proximal to distal to the innervation site. After tracing the nerves down to the muscle, any excess fat and fascia were removed from the subscapularis muscle to identify the myotendinous junction. The distance from the myotendinous junction of the subscapularis muscle to the insertion of upper, lower, and accessory nerve to the muscle was recorded while the shoulder was externally rotated.

## 3. Results

**Figure 1:**
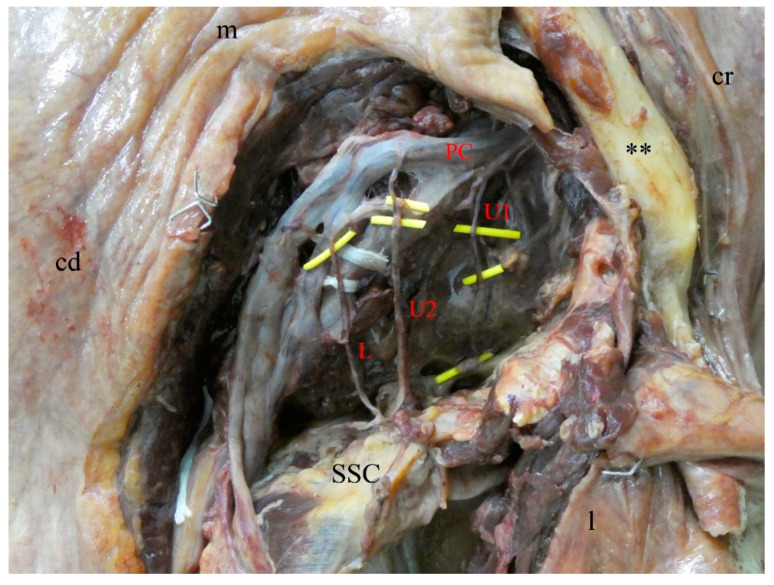
Left shoulder area of a 79-year-old male. The pectoralis major is removed. The brachial plexus is everted to view the full expansion of the subscapular nerves. The three subscapular nerves (U1, U2, L) are highlighted in yellow. U1, first upper subscapular nerve; U2, second upper subscapular nerve; L, lower subscapular nerve; PC, posterior cord of the brachial plexus; SSC, subscapularis muscle; **, clavicle; cd, caudal; cr, cranial; l, lateral; m, medial.

**Figure 2:**
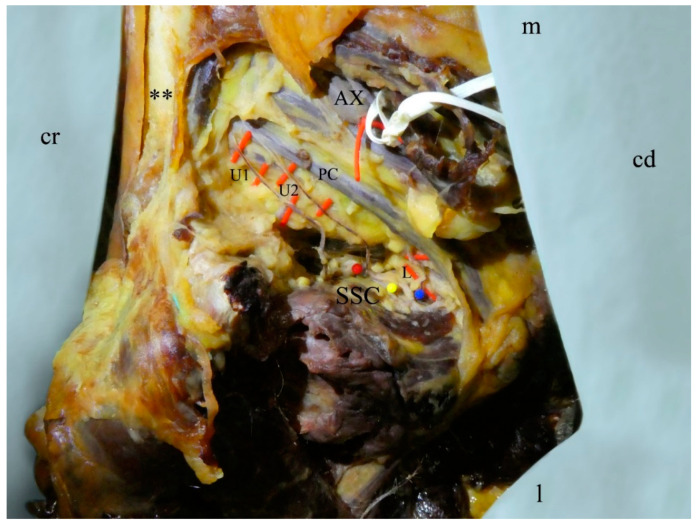
Right shoulder area of a 90-year-old female, anterolateral view. The pectoralis major is removed. The three subscapular nerves (U1, U2, L) are highlighted in red. The three dots (red, yellow, blue) mark the insertion points of the subscapular nerves into the muscle. U1, first upper subscapular nerve; U2, second upper subscapular nerve; L, lower subscapular nerve; PC, posterior cord of the brachial plexus; SSC, subscapularis muscle; AX, axillary artery; **, clavicle; cd, caudal; cr, cranial; l, lateral; m, medial.

An accessory third subscapular nerve was observed in three out of 31 cases: one male left side (Figure 1) and one male and female right side (Figure 2). These accessory nerves originated separately, proximally to the thoracodorsal nerve, from the posterior cord of the brachial plexus, and terminated in the upper and middle part of the subscapularis muscle. Based on these two criteria, the given three nerve branches were identified as accessory upper subscapular nerves.

The distance from the myotendinous junction to the insertion points of the subscapular nerves in two specimens with an accessory subscapular nerve is shown in Table 1. The distances for the usual configuration with one upper and one lower subscapular nerve range from 24 to 56 mm for the upper and 24 to 53 mm for the lower subscapular nerve.

## 4. Discussion

The purpose of this study was to investigate the frequency of accessory subscapular nerves. Such variations need to be considered during various surgical procedures to minimize the risk of iatrogenic injury.

Among the existing studies on the anatomy of the subscapular nerves, only few reports exist on accessory subscapular nerves. The frequency of the here-described accessory nerves varies from 7.4% [10] to 48.5% [14] for a single accessory upper subscapular nerve, 0% [8,10] to 6.1% [14] for two accessory upper subscapular nerves, and 0% [12] to 27.3% [14] for a single accessory lower subscapular nerve.

In our case series, we found an accessory upper subscapular nerve in 3 of 31 shoulders (9.7%), similar to studies published elsewhere [15].

In Table 2 the number of reported upper and lower subscapular nerves are shown.

Sager and colleagues [4] measured the distance from the insertion of the upper and lower subscapular nerve to the myotendinous junction with the arm adducted and externally rotated, in neutral position and internally rotated. For external rotation the distance to the upper subscapular nerve was 53.0 ± 14.7 mm (range 32–85 mm) and to the lower subscapular nerve 45.5 ± 13.8 mm (range 26–71 mm). Comparing these numbers to our own measurements it can be said that the accessory nerves fall into the range of the upper as well as the lower subscapular nerve. Therefore, it cannot be deduced just from distance measuring whether accessory nerves are specific and accessory upper or lower nerves. According to the small number of specimens with accessory subscapular nerves in our own dissection, it is unclear whether the insertion points of the accessory upper nerves tend to be in proximity to the typical upper nerves. This warrants further investigation with a larger number of specimens to observe more specimens with accessory nerves.

Based on the existing literature, the origin of the subscapular nerves seems rather variable. Even though some authors report that the branching pattern of the upper [8,9] and lower [8,9,12] subscapular nerves found in anatomy textbooks with both nerves originating from the posterior cord is not the most common version, as shown in Table 3 and Table 4 overall the most common origin for both the upper and lower subscapular nerve is the posterior cord of the brachial plexus.

Nevertheless, these findings apply to individual nerves and the frequency of a combination of the origin of both nerves cannot be deduced from these results.

The variability of the branching pattern of the subscapular nerve, as well as the frequency of accessory subscapular nerves needs to be considered especially during axillary dissection (e.g., lymphadenectomy) as during this procedure the thoracodorsal nerve is exposed [19] and any originating subscapular nerves are at risk of (iatrogenic) injury.

When approaching the shoulder joint anteriorly, the subscapularis muscle may have to be tenotomized, removed from the lesser tubercle or split in line with the muscle fibers [20,21]. This needs to be done among other procedures for shoulder arthroplasty and exposure of the glenoid during a Latarjet procedure. The subscapular nerves are in proximity to the operating field while approaching the shoulder anteriorly [16,22] and therefore are at risk to be sectioned, especially when splitting the muscle in line with its fibers. Furthermore, the nerves may be damaged because of incidental compression with retractors. Therefore, it is important to know the course of the subscapular nerves, the location of the entrance points, and the fact that variations and accessory nerves are not uncommon.

For plastic and reconstructive surgeons, orthopedics and neurosurgeons, the variability introduced by the branching pattern of the brachial plexus and the subscapular nerves seems highly relevant [23], especially if the lower subscapular nerve is used (e.g., for reconstruction of the axillary nerve) [24]. Furthermore, in painful hemiplegic shoulders, nerve blocks of the subscapularis used to improve the shoulder mobility may likewise benefit from our findings presented here [25].

## 5. Limitations

The number of tissues included in our own dissections was limited. The origins of the subscapular nerves were only documented for the cases with occurring accessory nerves.

Furthermore, because of the postmortem delay and effects introduced by the anatomical fixation caused by tissue acellularization [18], there might be a bias in the number of branches reported in our case series.

## 6. Conclusions

In dissection, single accessory upper subscapular nerves in 9.7% and no accessory lower subscapular nerves were observed. Overall, in the existing literature single accessory subscapular nerves were observed in 11.5% of the upper and 5% of the lower subscapular nerves. Double accessory subscapular nerves were observed less frequently. These findings warrant further investigations to be applied in a clinical setting.

## Figures and Tables

**Table 1:** Distance of the insertion points of the subscapular nerves to the myotendinous junction.

Nerve	Specimen 1	Specimen 2
Upper 1	44 mm	48 mm
Upper 2	37 mm	43 mm
Lower	39 mm	33 mm

**Table 2:** Summary of the numbers of accessory subscapular nerves. Numbers next to the authors give the total of specimens included in the respective studies.

	Single Accessory Upper	Double Accessory Upper	Single Accessory Lower	Double Accessory Lower
Ballesteros and Ramirez (*n* = 57)	18 (31.6%)	0	4 (7%)	0
Kasper et al. (*n* = 20)	4 (20%)	0	3 (15%)	0
Leischinger et al. (*n* = 20)	2 (10%)	0	4	0
Muthoka et al. (*n* = 68)	5 (7.3%)	1 (14.7%)	0	0
Saleh et al. (*n* = 33)	16 (48.5%)	2 (6.1%)	9 (27.3%)	1 (3%)
Tubbs et al. (*n* = 62)	5 (8.1%)	1 (1.6%)	4 (6.5%)	0
Young et al. (*n* = 11)	5 (45.5%)	0	0	0

**Table 3:** Summary on the various innervation patterns of the upper subscapular nerve. Numbers next to the authors give the total of specimens included in the respective studies.

	Posterior Cord	Axillary Nerve	Thoracodorsal Nerve	Posterior Division of Superior Trunk	Others/Not Specified
Ballesteros and Ramirez (*n* = 57)	19 (33.3%)	3 (5.3%)		28 (49.1%)	7 (12.3%)
Chaudhary et al. (*n* = 60)	55 (91.7%)			5 (8.3%)	
Fazan et al. (*n* = 54)	19 (35.1%)	3 (5.6%)		32 (59.3%)	
Kasper et al. (*n* = 20)	14 (70%)				6 (30%)
Leischinger et al. (*n* = 20)	20 (100%)				
Muthoka et al. (*n* = 68)	56 (82.4%)	10 (14.7%)			2 (2.9%)
Rastogi et al. (*n* = 74)	74 (100%)				
Sager et al. (*n* = 20)	20 (100%)				
Saleh et al. (*n* = 33)	33 (100%)				
Tubbs et al. (*n* = 62)	60 (96.8%)	2 (3.2%)			
Young et al. (*n* = 11)	11 (100%)				
Total	381 (79.5%)	18 (3.8%)		65 (13.6%)	15 (3.1%)

**Table 4:** Summary on the various innervation patterns of the lower subscapular nerve. Numbers next to the authors give the total of specimens included in the respective studies.

	Posterior Cord	Axillary Nerve	Thoracodorsal Nerve	Posterior Division of Superior Trunk	Others/Not Specified
Ballesteros and Ramirez (*n* = 57)	18 (31.6%)	31 (54.4%)	7 (12.2%)		1 (1.8%)
Chaudhary et al. (*n* = 60)	58 (96.7%)	2 (3.3%)			
Fazan et al. (*n* = 54)	21 (38.9%)	29 (53.7%)	4 (7.4%)		
Kasper et al. (*n* = 20)	13 (65%)	5 (25%)	2 (10%)		
Leischinger et al. (*n* = 20)	14 (70%)	4 (20%)	2 (10%)		
Muthoka et al. (*n* = 68)	12 (17.7%)	43 (63.2%)	9 (13.2%)		4 (5.9%)
Rastogi et al. (*n* = 74)	74 (100%)				
Sager et al. (*n* = 20)	17 (85%)	3 (15%)			
Saleh et al. (*n* = 33)	27 (81.8%)	5 (15.2%)	1 (3%)		
Tubbs et al. (*n* = 62)	49 (79%)	13 (21%)			
Young et al. (*n* = 11)	7 (63.6%)	1 (9.1%)	3 (27.3%)		
Total	310 (64.7%)	136 (28.4%)	28 (5.8%)		5 (1.1%)
